# Assessment of magnetic resonance image compilation (MAGiC) abilities of therapeutic selection and prediction on recurrence risk factors and short-term treatment efficacy in cervical cancer

**DOI:** 10.1007/s11547-025-02042-7

**Published:** 2025-07-04

**Authors:** Xiaorong Ou, Hui Liu, Weiyin Vivian Liu, Yue Li, Wenguang Liu, Yu Bai, Jinbiao Chen, ZhengHao Deng, Wenzheng Li, Yigang Pei

**Affiliations:** 1 Department of Radiology, Xiangya Hospital, Central South University, Changsha, 410008 Hunan People’s Republic of China; 2 National Clinical Research Center for Geriatric Disorders, Xiangya Hospital, Central South University, Changsha, 410008 Hunan People’s Republic of China; 3 MR Research, GE Healthcare, Beijing, People’s Republic of China; 4 Department of Medical Records and Information, Xiangya Hospital, Central South University, Changsha, 410008 Hunan People’s Republic of China; 5 Department of Pathology, Xiangya Hospital, Central South University, Changsha, 410008 Hunan People’s Republic of China

**Keywords:** Magnetic resonance imaging, Uterine cervical neoplasms, Patient care planning, Risk factors, Treatment outcome

## Abstract

**Objectives:**

To investigate the feasibility of MAGiC (hereafter, sy-T2WI; T1, T2, and PD maps) in determination of treatment plan and prediction of recurrence risk factors (RRF) and short-term treatment efficacy (STE) in patients with cervical cancer (CC) using hr-T2WI and DWI as reference standards.

**Methods:**

119 consecutive CC patients who underwent MAGiC, hr-T2WI and DWI were prospectively recruited from October 2021 to March 2024. The subjective evaluation of image quality and tumor staging using sy-T2WI and hr-T2WI was conducted. The accuracy, sensitivity and specificity of sy-T2WI were analyzed for selection of a treatment strategy (stage IB-IIA: surgical operation; stage IIB-IVA: CCRT). RRF and STE were evaluated in staging IB-IIA and IIB-IVA CC patients respectively. Then, the area under the curve (AUC) was used to objectively predict RRF and STE using the quantitative T1, T2, PD maps, their combinations, and apparent diffusion coefficient (ADC).

**Results:**

There was no significant difference of image quality (all *P* > 0.05), but a strong agreement on tumor staging (Kappa value = 0.935; *p* < 0.001) between sy-T2WI and hr-T2WI. The accuracy, sensitivity, and specificity of sy-T2WI in deciding treatment strategies were 0.908, 0.908, and 0.999, respectively. Furthermore, the combination of T1 and T2 values was superior to ADC values for predicting RRF (AUC: 0.980 vs. 0.776; *p* = 0.005) in staging IB-IIA and STE (AUC: 0.982 vs. 0.737; *p* < 0.001) in IIB-IVA CC subjects.

**Conclusions:**

MAGiC is a promising technique for determination on treatment selection, RRF prediction and STE prognosis in CC patients as its performance is equivalent and even superior to hr-T2WI and DWI.

**Supplementary Information:**

The online version contains supplementary material available at 10.1007/s11547-025-02042-7.

## Introduction

Cervical cancer (CC) is the fourth most common cancer and the leading cause of cancer-related death among women worldwide [1]. The mortality rate of CC in China has been on the rise in recent years [2]. Treatment selection is determined by the International Federation of Gynecology and Obstetrics (FIGO) tumor stage: surgical resection for FIGO stage IB-IIA (early-stage CC) [3] and concurrent chemoradiotherapy (CCRT) for stage IIB–IVA tumors (locally advanced CC) [4].

Recurrence risk factors (RRF) such as lymph node metastasis (LNM) and positive lymphovascular space invasion (LVSI) increase the likelihood of recurrence. In other words, recurrence is reduced if an early-stage CC patient determined with RRF received additional postoperative adjuvant radiotherapy or cisplatin-based chemotherapy after surgical resection [5–8]. Local recurrence or distant metastasis develop in approximately 30% of CCRT-treated patients with locally advanced CC when poor treatment response presents [9]. Therefore, tumor stage, therapeutic response and RRF dominate individualized therapy efficacy.

Pelvic MR imaging, such as high-resolution T2-weighted imaging (hr-T2WI) and diffusion-weighted imaging (DWI), has been widely utilized to assess tumor stage and treatment responses as well as predict recurrence [10]. hr-T2WI as a morphologic imaging can qualitatively assists a radiologist to accurately identify cervical tumor and invasion extension to the uterus, parametrium and adjacent organs [11, 12] and shows good diagnosis performance on tumor stage with reported accuracy of 90% for early-stage tumors (IB-IIA) [13] and 89% for locally advanced tumors (IIB–IVA) [14]. On the other hand, DWI-derived apparent diffusion coefficient (ADC) as a functional imaging quantitatively reflects RRF of cervical cancer and has good prediction efficacy for LNM and LVSI respectively with area under the curve (AUC) of 0.74–0.79 [15] and 0.767−0.950 [16]. Additionally, it moderately reflects therapeutic response in CCRT-treated CC patients [17–19] with sensitivity and specificity of 73% and 72.5%, respectively [20]. Therefore, hr-T2WI and DWI are reliable in terms of tumor staging as well as predicting RRF and therapeutic response.

In clinical practice, single acquisition for simultaneously qualitative and quantitative diagnosis of cervical tumors may consume less scan time. Recently, MAGnetic resonance imaging Compilation (MAGiC) carried out using a two-dimensional fast spin echo multi-delay multi echo (MDME) technique and generates multiple contrast images as well as quantitative maps (T1, T2 and PD maps) in one scan within a clinically-acceptable scan time for diagnosis of brain, prostate, and rectal diseases [21–27]. Notably, MAGiC offers both morphologic (synthetic T2WI, sy-T2WI) and quantitative (T1, T2, and proton density (PD) maps) images in a single acquisition. Therefore, this study aimed to (1) evaluate the image quality of morphologic MAGiC (sy-T2WI) in comparison with hr-T2WI, (2) explore the feasibility of sy-T2WI in tumor staging in comparison with hr-T2WI as a standard reference, (3) investigate the inter-patient variability of MAGiC-generated T1, T2, and PD maps, with a particular focus on assessing the predictive accuracy of radiomic risk factors (RRFs) for early-stage CC patients and short-term efficacy (STE) prognosis following CCRT for locally advanced CC patients using T1, T2, PD maps in comparison with DWI.

## Materials and methods

### Study participant

**Fig. 1:**
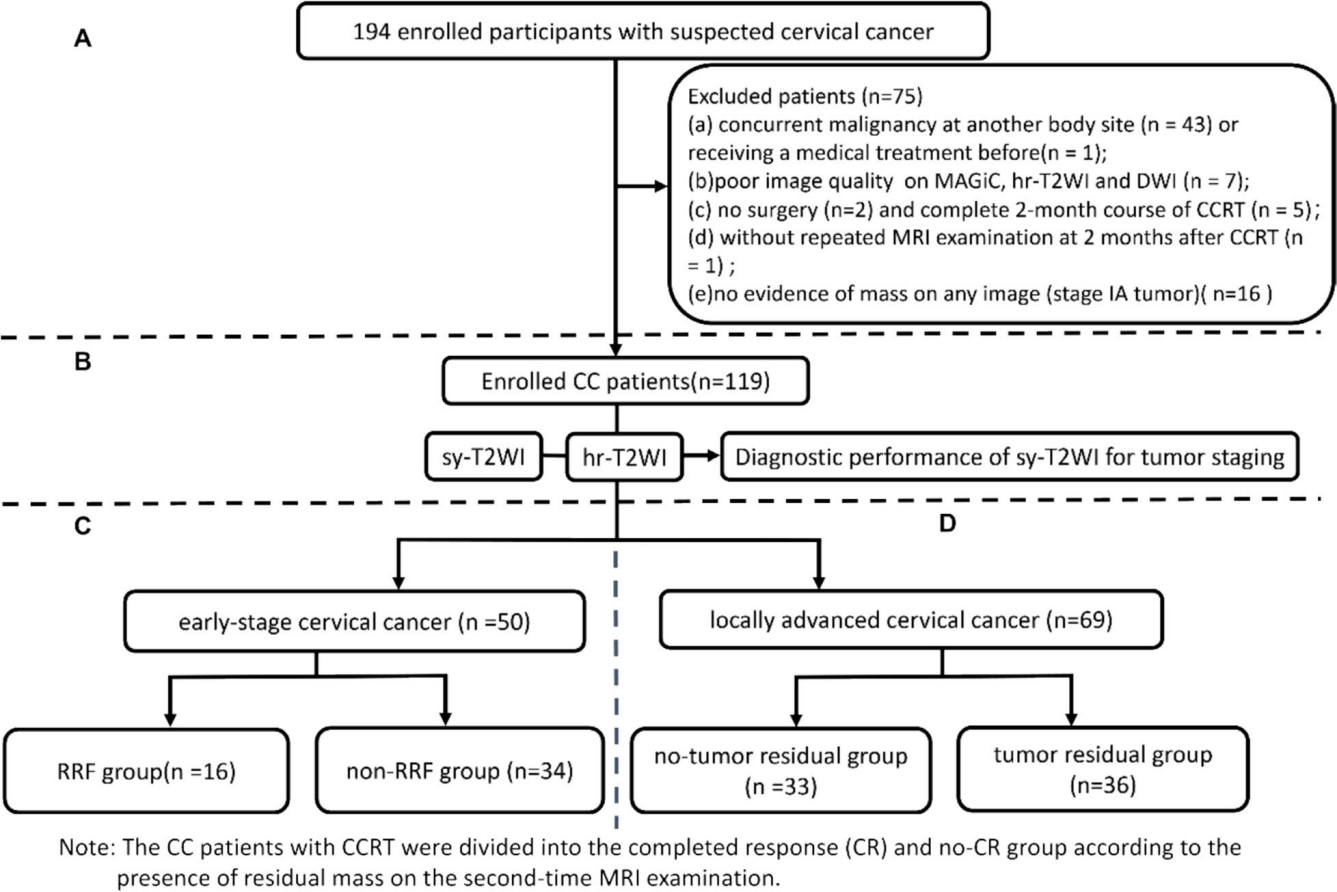
This study flowchart shows the participant enrollment, tumor staging, and RRF prediction and STE evaluation

This study was approved by the institutional review board of our hospital. A total of 194 patients and signed an informed consent form and then prospectively and consecutively enrolled our study between October 2021 and March 2024. Inclusion criteria were: (a) age 18 years or older; (b) histologically-confirmed cervical cancer, including squamous cell carcinoma and adenocarcinoma with various tumor differentiation grades; (c) no previous pelvic therapy (e.g. chemoradiotherapy, concurrent chemoradiotherapy or surgical resections before MRI examination); (d) scanned MAGiC, hr-T2WI, and DWI within 10 days before treatment; and (e) a surgery or CCRT on schedule. The exclusion criteria were: (a) concurrent malignancy at another body site or receiving a medical treatment before; (b) poor image quality of any one image data set (MAGiC, hr-T2WI, and DWI); (c) no surgery or complete 2-month course of CCRT; (d) without repeated MRI examination at 2 months after CCRT; (e) no evidence of mass on any image (such as stage IA tumor). Screening according to the inclusion and exclusion and those suitable CC patients were statistically analyzed in this study (Fig. 1A–D).

### MRI examination

All pelvic MR examinations including MAGiC, hr-T2WI (oblique axial, sagittal, and coronal views), dynamic contrast-enhanced MRI (DCE-MRI), oblique axial Multiple Sensitivity Encoding Diffusion-weighted imaging (MUSE-DWI) were performed on a 3.0 T MR scanner (Signa Premier, GE Healthcare, Waukesha, WI, USA) using a 32-channel body array coil. MAGiC and DWI were acquired in the same axial plane (28 slices), slice thickness (3.5 mm), and spacing (0.5 mm) as the oblique axial hr-T2WI (perpendicular to the cervix). The other detailed scan parameters are summarized as follow: The sequence parameters of MAGiC were performed without fat suppression technique, repetition time (TR) = 4000 ms, echo time (TE) = 16 ms for TE1, 91.6 for TE2, bandwidth (BW) = 50 Hz/pixel, field of view (FOV) = 34 cm, number of excitations (NEX) = 1, matrix = 320 × 256, in-plane resolution = 1.1 mm × 1.3 mm, Acceleration = 2.0; hr-T2WI were carried out with fast spin echo (FSE) without fat suppression technique, TR = 3573 ms, TE = 110 ms, BW = 62.5 Hz/pixel, FOV = 20 cm, matrix = 332 × 332, in-plane resolution = 0.6 mm × 0.6 mm, Flip angle = 111°, Acceleration = 2.0; MUSE-DWI were scanned with fat suppression technique, TR = 3000 ms, TE = 65.1 ms, FOV = 26 mm, NEX = 2, 4, matrix = 216 × 216, in-plane resolution = 1.2 mm × 1.2 mm, b value = 0, 800 s/mm2, Acceleration = 2.0. The MAGiC sequence requires approximately 4 min and 32 s of scan time compared to the integrated utility of hr-T2WI (3 min 8 s) and MUSE-DWI (2 min 32 s). Importantly, despite its slightly longer acquisition duration, MAGiC significantly enhances the workflow efficiency and patient comfort while providing clinically valuable T1/T2/PD quantitative values that improve diagnostic confidence for radiologists.

**Fig. 2:**
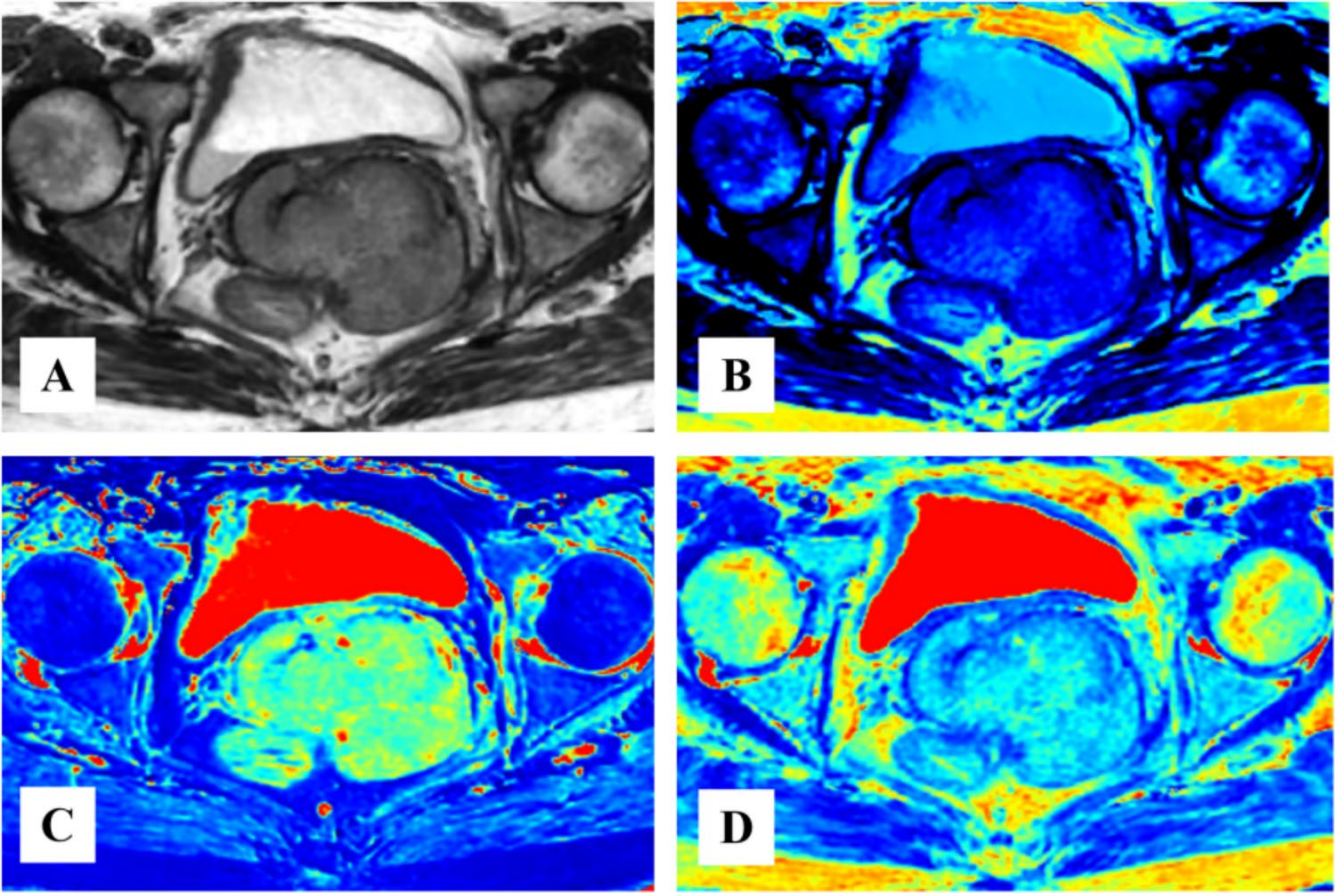
Representative synthetic magnetic resonance images (MAGiC) of a 38-year-old female with cervical cancer includes **A** synthetic T2WI (sy-T2WI), **B** T1 map, **C** T2 map, and **D** PD map, respectively

Overall, one-scan MAGiC took less about 1 min than hr-T2WI and MUSE-DWI. In addition, sagittal and coronal hr-T2WI and axial dynamic contrast enhanced MRI were also performed for each CC patient. Synthetic morphologic (synthetic T2WI, sy-T2WI) and quantitative images (T1, T2 and PD maps) were obtained using MAGiC software (MAGiC, v. 100.1.1, Sy-MRI 7.2) (Fig. 2A–D) while apparent diffusion coefficient (ADC) maps were also generated from MUSE-DWI with b values of 0 and 800 s/mm^2^ on the scanner console. DCE-MRI was performed using a dose of 0.2 ml/kg extracellular contrast agent (gadodiamide, Omniscan, GE Healthcare) followed by 20 ml of 0.9% saline at an injection rate of 2 ml/s. All image post-processing were obtained on Advanced Workstation (AW 4.7, GE Healthcare).

## Synthetic morphologic images

### Image quality

The image quality of sy-T2WI and hr-T2WI were independently assessed by two radiologists (Reader 1 and 2 with 5 and 3 years of experience in cervical MRI, respectively) in a consensus. A 4-point scale was used to assess the image quality, including (a) anatomical details (1 = poor, 2 = fair, 3 = good, 4 = excellent), (b) distortion (1 = severe, 2 = moderate, 3 = slight, 4 = absent), (c) artifacts (1 = serious, 2 = moderate, 3 = slight, 4 = absent) and (d) lesion conspicuity (1 = poor, unrecognized, 2 = fair, mostly unclear outlines, 3 = good, partly unclear outlines, 4 = excellent, clear outlines) [28]. An inter-observer agreement on image quality scores were analyzed between sy-T2WI and hr-T2WI.

### Tumor stage

**Fig. 3:**
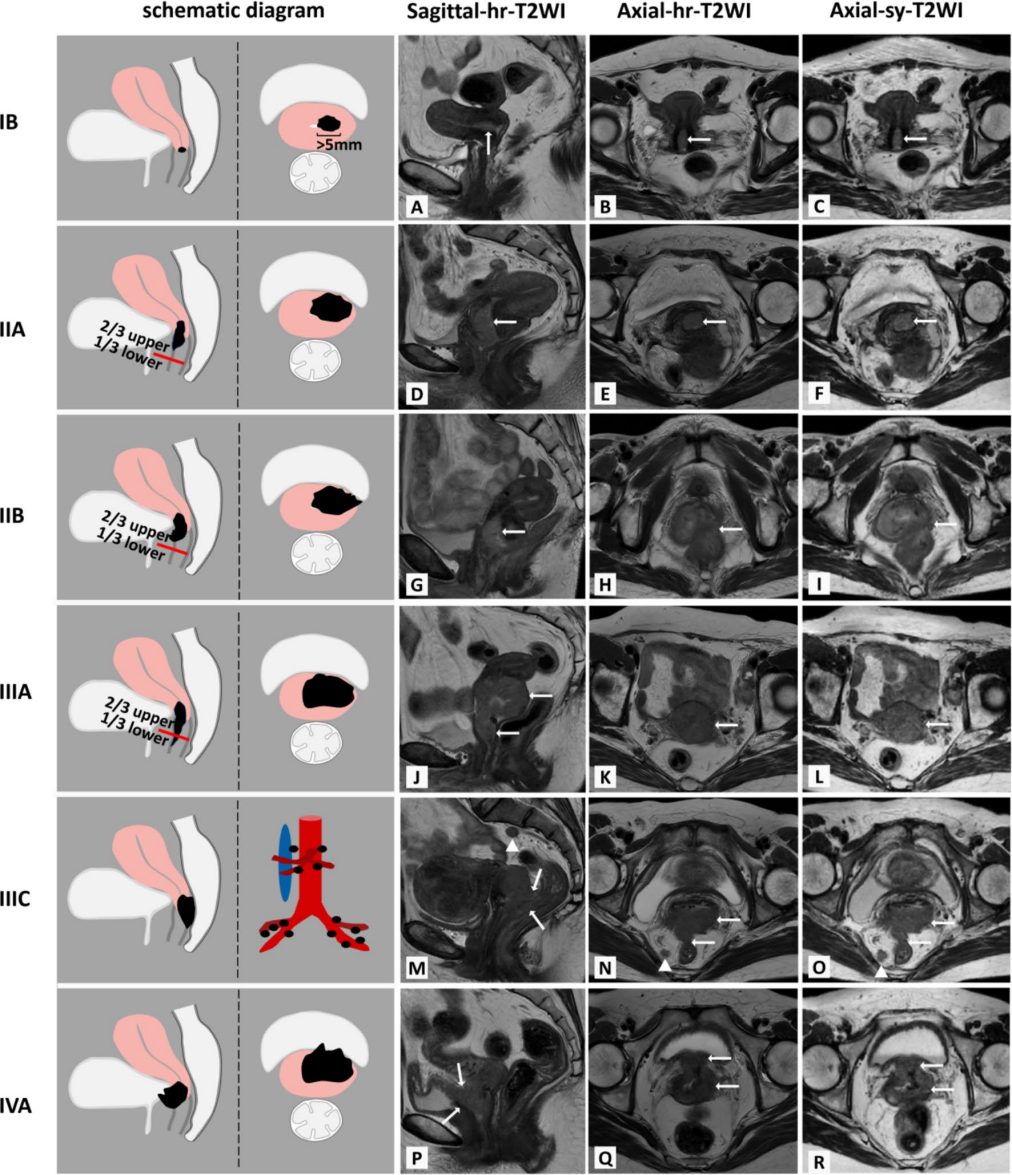
The evaluation of tumor stage (IB-IVA) with synthetic T2WI (sy-T2WI; the last column) compared to conventional T2WI (hr-T2WI; the fourth column) using the 2018 FIGO criterion. **A–C** The size of tumor stage IB (arrow) is about 7 mm (> 5 mm) in the greatest dimension. **D–F** The stage IIA mass (arrow) is limited to 2/3 of the vaginal wall without perimetrial invasion. **G–I** The stage IIB mass (arrow) shows perimetrial invasion. **J–M** shows stage IIIA tumor invading (arrow) the lower 1/3 of the vagina, but with no extension into the pelvic wall. Figure **N** and **O** depicts a stage IIIC tumor with positive lymph nodes in the pelvic and/or paraaortic (triangle) (arrow). Figure **P–R** shows stage IVA mass (arrow) invading adjacent organs(bladder)

Two radiologists (Reader 1 and 2) were blind to the patients’ clinical and pathological information and evaluate tumor stage in a consensus according to 2018 FIGO criterion including tumor size, vaginal or perimetrial involvement, bladder/rectum extension, and distant metastases (Fig. 3). The assessment was performed on the sy-T2WIs in one week and hr-T2Wis in another week [9, 10] (Fig. 3). Patients with biopsy-confirmed CC were classified into stage IA even if there is no visible lesion with/without maximum invasive depth ≤ 5 mm to the eyes on the morphological images [3].

 The accuracy (Acc), sensitivity (Sen), and specificity (Spec) of sy-T2WI in differentiating stage IB-IIA from stage IIB-IVB were determined using Chi-square (χ2), using hr-T2WI as the reference standard (Fig. 1B). The inter-modality agreement was also examined using a weighted kappa coefficient (*k*).

### Recurrence risk factors

All patients with diagnosed FIGO stage IB-IIA based on hr-T2WI underwent surgical resection. Pathological examination was performed by a 30-year-experienced pathologist who was blind to the patient's clinical data and image analysis. The tumor characteristics including recurrence risk factors (RRF), histopathologic grade, tumor and node stage were evaluated according to the 2018 FIGO staging system and reported using the standard pathological template.

The evaluation of RRF included: (1) lymph node metastasis (LNM), degined as malignant epithelial cells invasion into the lymph node [29] (Supplementary Fig. 1A); (2) positive lymphovascular space invasion (LVSI), characterized by the tumor cells present within endothelial-lined lymphatic vessels without musclar wall involvement [30] (Supplementary Fig. 1B); (3) deep cervical stromal invasion (DSI), indicating tumor infiltration exceeding one-third of the cervical wall thickness [30] (Supplementary Fig. 1C); (4) tumor maximum diameter (TMD) greater than 4 cm [3] (Supplementary Fig. 1D). Based on postoperative pathological findings, patients were stratified into RRF (presence of ≥ 1 risk factor) and non-RRF groups (absence of all factors) groups (Fig. 1C), enabling comparative anaylsis between these clinically distinct cohorts.

### Follow-ups for evaluation of STE

All patients with stage IIB–IVA tumor based on hr-T2WI received 5 cycles of CCRT (40 mg/m^2^ of weekly cisplatin and intensity-modulated radiation therapy; a total dose of 45–50 Gy). Two months after CCRT, a repeated hr-T2WI, DWI and DCE-MRI were performed for evaluation of treatment response as the short-term efficacy (STE).

The existence of residual mass was determined by two senior radiologists (Readers 3 and 4, with 15 and 20 years of experience in pelvic MRI, respectively) in a consensus based on hr-T2WI (persistent tumor), DCE-MRI (an enhanced area) and DWI (a hyperintense area) at 2 months after CCRT [11] and then also grouped into the residual group and the non-residual group (Fig. 1D).

### Synthetic quantitative images

**Fig. 4:**
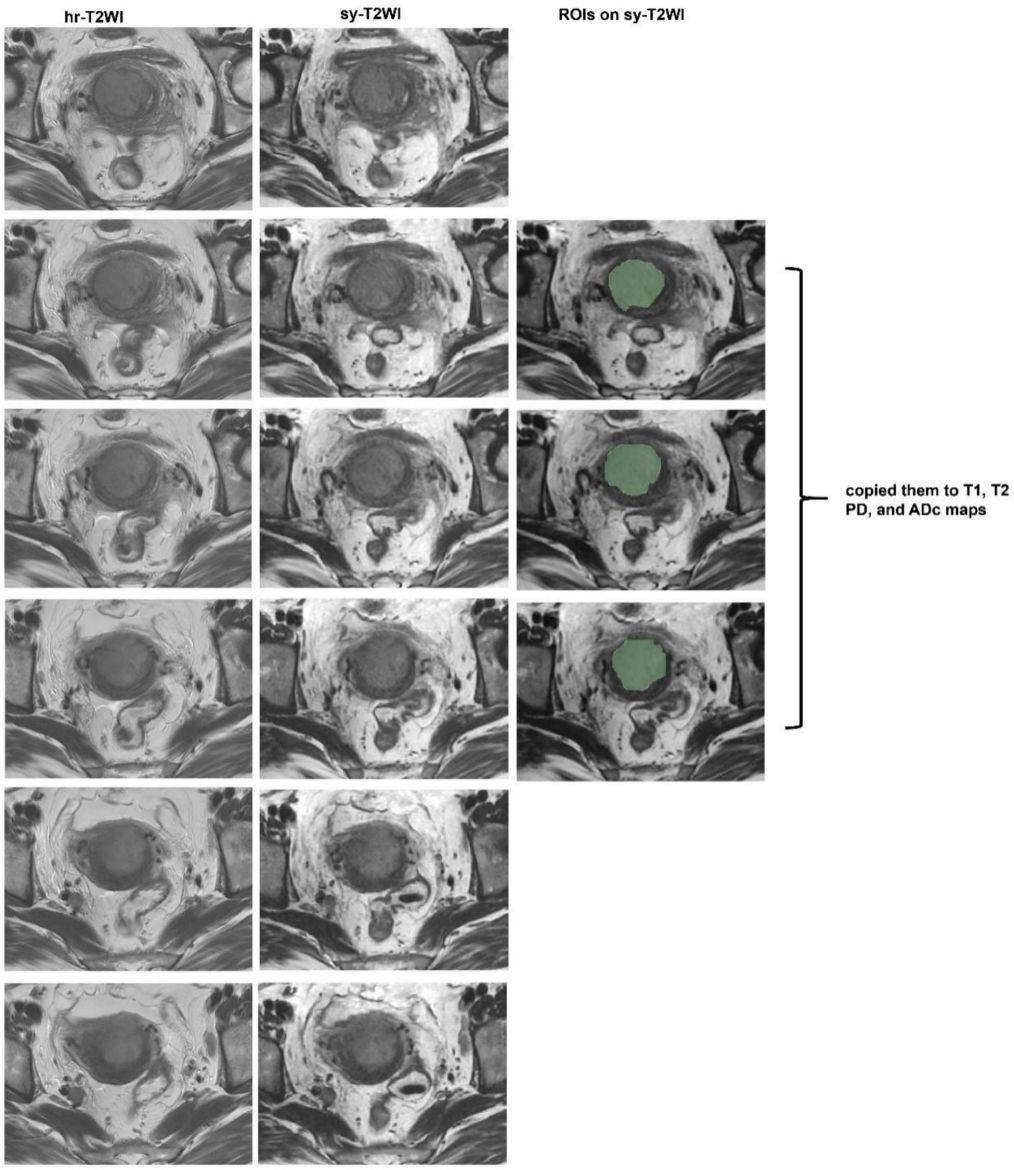
The schematic diagram depicts three-slice region of interests (ROIs) sketched on sy-T2WI. The last column shows the manually sketched ROIs at three slices of the mass on sy-T2WI, including the slice with the largest mass area and its upper and lower slices. ROIs were copied to T1, T2, PD and ADC maps to obtain the corresponding T1, T2, PD and ADC values for each CC patient. The corresponding sy-T2WI (the second column) and hr-T2WI (the first column) are also shown

Firstly, the size, extent, and boundary of the lesions were identified on sy-T2WI and DWI. A senior radiologist with over 30 years of experience in gynecologic MR imaging determined the final diagnosis when there was a disagreement between the radiologists. Three-slice region of interests (ROIs) were manually drawn on consecutive slices of sy-T2WI by Readers 1 and 2 along the margins of the residual lesions and avoiding areas of necrosis, including the slice with the largest mass area and its upper and lower slices. In addition, a region of interest (ROI) was also sketched on the anterior wall of normal myometrium to validate the inter-patient variability by Readers 1 and 2. Next, the ROIs were copied to T1, T2, PD, and ADC maps to obtain the corresponding value (mean value) of T1, T2, PD, and ADC (Fig. 4). In our study, average ROI size was 5.97 ± 3.54 cm^2^ (0.41–14.77 cm^2^) for tumor and 2.42 ± 1.83 cm^2^ (0.97–5.1 cm^2^) for myometrium.

### Statistical analyses

Statistical analysis was performed using SPSS Statistics version 27.0 (IBM Corp., Armonk, N.Y., USA) and GraphPad (version 9.0.1). The image quality scores (including anatomical details, distortion, artifacts and lesion conspicuity) for morphologic sy-T2WI and hr-T2WI were expressed as median [minimum, maximum] and analyzed for the inter-observer agreement using the weighted kappa coefficient (*k*, < 0.20, poor repeatability; 0.21–0.40, fair; 0.41–0.60, moderate; 0.61–0.80, moderate; > 0.80, excellent or strong reliability). In addition, the quantitative mean T1, T2, PD and ADC values of myometrium and tumor were compared to distinguish RRF from non-RRF in early-stage and tumor residual group from non-tumor residual in locally advanced cervical cancer using a paired t test. Furthermore, receiving operation characteristics (ROCs) for T1, T2, and PD maps to predict STE, differentiation of tumor residual and non-residual groups, were plotted for cut-off retrieval and the DeLong test was used to compare the area under the curve (AUC) and 95% confidence intervals (CI) of the quantitative maps in comparison with an ADC map. Furthermore, the combination model (composed by the significal T1, T2 and PD) were analyzed. Significant differences were set at *P* values less than 0.05.

## Results

### Clinical characteristics

A total of 119 (mean age: 50.4 ± 10.0 years; age range: 26–80 years; staging IB: 31; IIA:19; IIB: 26; IIIA: 8; IIIB: 4; IIIC: 25; IVA:6) out of the 194 CC patients were enrolled, including 50 subjects (mean age: 52.4 ± 9.2 years; age range: 26–84 years) with early-stage CC (staging IB-IIA) for surgical resection and 69 cases (mean age: 54.6 ± 10.2 years; age range: 36–86 years) with locally advanced CC (staging IIB-IVA) for CCRT and the two-month follow-up. All early-stage CC patients were divided into RRF group (N = 16 [LVSI: 9, LNM + LVSI: 2, LVSI + TMD:1, LVSI + DSI: 4], mean age: 44.4 ± 4.7 years; age range: 26–64 years) and non-RRF group (N = 34, mean age: 57 ± 6.3 years; age range: 37–84 years) according to the histological examination. All locally advanced CC patients were classified into tumor residual group (N = 36, mean age: 53.4 ± 5.6 years; age range: 35–64 years) and non-residual group (N = 33, mean age: 57 ± 8.3 years; age range: 37–86 years) after CCRT. The patients’ clinical characteristics are detailed in Supplementary Table 1, including squamous cell carcinoma (n = 102) and adenocarcinoma (n = 17), with tumor differentiation grades distributed as well/moderately differentiated (n = 104) and poorly differentiation tumors (n = 14).

### Synthetic morphologic images

**Fig. 5:**
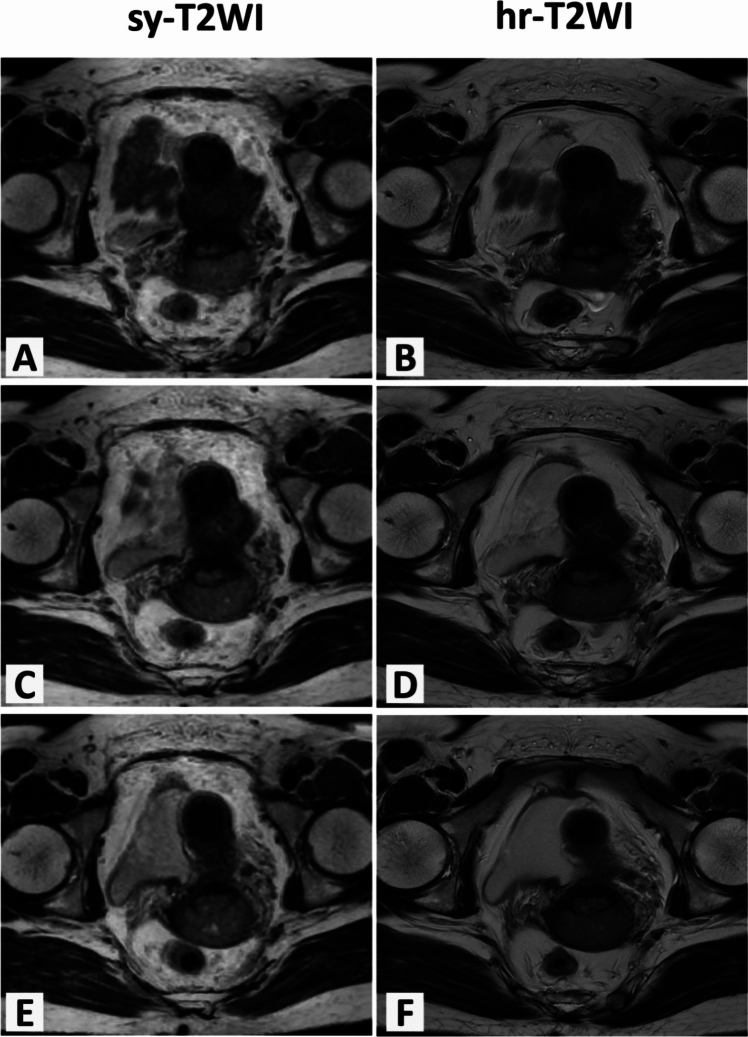
Comparisons of image quality of synthetic T2WI (sy-T2WI; the first column) and conventional T2WI (hr-T2WI; the second column) in a 53-year-old female with IIB cervical cancer. The image quality of sy-T2WI was similar to hr-T2WI regarding the anatomical details (4 vs. 4), distortion (4 vs. 4), artifacts (4 vs. 4) and lesion conspicuity (4 vs. 4) with a 4-point scale on Figure (**A**)–(**F**)

In 119 CC patients, there was no significant different scores between sy-T2WI and hr-T2WI regarding anatomical details, distortion, artifacts, and lesion conspicuity of the tumor (all *P* > 0.05; Table 1, Fig. 5). Furthermore, the inter-observer agreement of rating scores was excellent for both sy-T2WI (k = 0.840–0.863, all *P* < 0.001) and hr-T2WI (k = 0.820–0.863, all *P* < 0.001) (Table 1). For tumor staging, the accuracy, sensitivity and specificity of sy-T2WI was 0.908, 0.908 and 0.999 for differentiating IB-IIA from IIB-IVA. There was an excellent agreement between sy-T2WI and hr-T2WI in distinguishing IB-IIA from IIB-IVA (k = 0.935; *p* < 0.001) (Table 2).

**Table 1:** Comparison of the image quality scores between sy-T2WI and hr-T2WI

	Sy-T2WI	Hr-T2Wl	P value (overall)	Inter-reader agreement for sy-T2WI	Inter-reader agreement for hr-T2WI
Anatomical detail					
Observer 1	4(3,4)	4(3,4)	0.529	0.841 (< 0.001*)	0.856(< 0.001*)
Observer 2	4(3,4)	4(3,4)	0.441		
Artifacts					
Observer 1	4(3,4)	4(3,4)	0.083	0.863 (< 0.001*)	0.820 (< 0.001*)
Observer 2	4(3,4)	4(3,4)	0.482		
Distortion					
Observer 1	4(3,4)	4(3,4)	0.783	0.840 (< 0.001*)	0.863 (< 0.001*)
Observer 2	4(3,4)	4(3,4)	0.250		
Lesion conspicuity					
Observer 1	4(3,4)	4(3,4)	0.207	0.853 (< 0.001*)	0.856 (< 0.001*)
Observer 2	4(3,4)	4(3,4)	0.253		

*p value indicates a significant difference; Median (min, max) values are expressed as the mean image quality 4-point rating scores for anatomical details display, distortion, artifacts and lesion conspicuity. The inter-observer agreement respectively on sy-T2WI and hr-T2WI was evaluated

**Table 2:** Comparison of tumor staging using sy-T2WI and hr-T2WI based on 2018 FIGO criterion

hr-T2WI	Sy-T2WI	The diagnostic agreement between sy-T2WI and hr-T2WI			The diagnostic performance of sy-T2WI using hr-T2WI as standard reference		
	IB-IIA	IIB -IVA	Kappa value	P value	Acc	Sen	Spec
IB-IIA	44	6	0.935	< 0.001	0.908	0.908	0.999
IIB -IVA	5	64					

Acc, accuracy; Sen, sensitivity; Spe, specificity

### Quantitative images for predicting RRF and STE

The mean T1, T2, ADC values of myometrium were not significant difference between RRF and non-RRF group as well as between tumor residual and non-residual group (all *P* > 0.05) (Table 3). For example, the average T1 values was similar in RRF and non-RRF group (1283.12 ± 48.76 ms vs.1304.01 ± 39.33 ms; *P* = 0.755) for early-stage cervical cancer patients (Table 3).

**Table 3:** Comparison of the quantitative mean T1, T2, PD and ADC values of myometrium and tumor for presenting the inter-patient variability, and distinguishing RRF from non-RRF in Early-stage, and CR group from non-CR group in Locally advanced cervical cancer

	The location of ROIs	Parameter	no-RRF (n = 34)	RRF (n = 16)	*p* value
Early-stage cervical cancer	myometrium	T1 (ms)	1304.01 ± 39.33	1283.12 ± 48.76	0.755
		T2 (ms)	82.97 ± 2.61	82.75 ± 3.94	0.963
		PD (p.u.)	83.18 ± 1.53	84.52 ± 3.46	0.727
		ADC (10^−6^mm^2^/sec)	1314.86 ± 38.74	1319.32 ± 49.03	0.946
	tumor	T1 (ms)	1288.80 ± 90.68	1180.40 ± 64.06	< 0.001*
		T2 (ms)	96.11 ± 2.78	91.00 ± 3.38	< 0.001*
		PD (p.u.)	86.16 ± 4.19	84.44 ± 3.74	0.168
		ADC (10^−6^mm^2^/sec)	994.72 ± 57.54	928.17 ± 52.83	< 0.001*

	The location of ROIs	Parameter	non-residual group (n = 33)	tumor residual group (n = 36)	*p* value
Locally advanced cervical cancer	myometrium	T1 (ms)	1236.82 ± 36.70	1283.27 ± 37.16	0.377
		T2 (ms)	83.59 ± 2.86	83.17 ± 2.42	0.910
		PD (p.u.)	83.40 ± 1.58	82.63 ± 1.84	0.751
		ADC (10^−6^mm^2^/sec)	1301.48 ± 34.56	1353.19 ± 40.72	0.339
	tumor	T1 (ms)	1134.19 ± 105.61	1273.93 ± 52.34	< 0.001*
		T2 (ms)	87.35 ± 1.87	90.76 ± 1.77	< 0.001*
		PD (p.u.)	79.90 ± 2.66	81.24 ± 3.24	0.065
		ADC (10^−6^mm^2^/sec)	939.82 ± 83.81	992.40 ± 55.46	0.003

ROI, region of interest; P.u., percentage unit; RRF, recurrence risk factors

*p value indicates a statistical difference

**Fig. 6:**
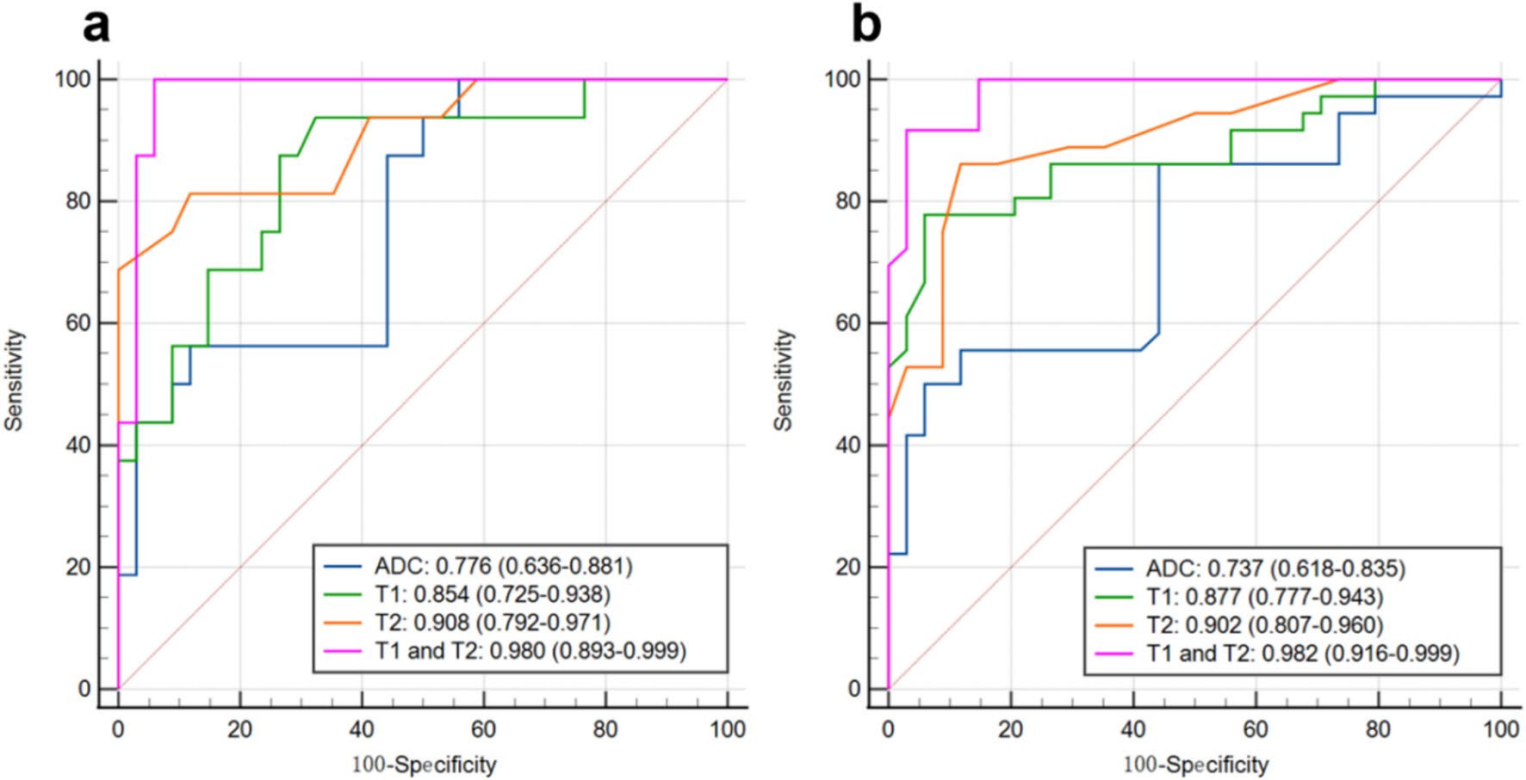
The diagnostic performance of T1, T2, their combination and ADC, models for predicting RRF and STE. **A** The predictive performance of ADC (AUC: 0.776, 95%CI: 0.636–0.881) was inferior to T1 + T2 (AUC: 0.980, 95%CI: 0.893–0.999; *p* = 0.005), which was similar with T1 (AUC: 0.854, 95% CI: 0.725–0.938; *P* = 0.338)) and T2 (AUC: 0.908, 95% CI: 0.792–0.971; *P* = 0.121) for distinguishing RRF from non-RRF in early-stage cervical cancer. **B** The predictive performance of ADC (AUC: 0.737, 95%CI: 0.618–0.835) was inferior to T1 + T2 (AUC: 0.982, 95%CI: 0.916, 0.999; *p* < 0.001) and T2 (AUC: 0.902, 95% CI: 0.807–0.960; *P* = 0.030), which was similar with T1 (AUC: 0.877, 95% CI: 0.777–0.943; *P* = 0.081) for predicting short-term efficacy in locally advanced cervical cancer with CCRT

Among patients with cervical cancer, the mean T1, T2, ADC values were significantly lower in the RRF group than the non-RRF group (T1: 1180.40 ± 64.06 ms vs. 1288.80 ± 90.68 ms; T2: 91.00 ± 3.38 ms vs. 96.11 ± 2.78 ms; ADC: 928.17 ± 52.83 × 10^–6^ mm^2^/sec vs. 994.72 ± 57.54 × 10^−6^mm^2^/sec; all *P* < 0.001) except for PD value (84.44 ± 3.74 p.u. vs. 86.16 ± 4.19 p.u.; *p* = 0.168) (Table 3). Furthermore, T1 and T2 values showed similar diagnostic performance on RRF prediction (AUC: 0.854, 95% CI: 0.725–0.938; AUC: 0.908, 95% CI: 0.792–0.971) to ADC value (AUC: 0.776, 95% CI: 0.636–0.881; all *P* > 0.05). The combination of T1 + T2 model (AUC: 0.980, 95%CI: 0.893–0.999) showed higher efficacy for RRF prediction than ADC alone (AUC: 0.980 vs. 0.776; *p* = 0.005) (Tale 4, Fig. 6).

For STE, the mean T1, T2 and ADC values were significantly lower in the non-residual group than in the tumor residual group (T1: 1134.19 ± 105.61 ms vs.1273.93 ± 52.34 ms; T2: 87.35 ± 1.87 ms vs. 90.76 ± 1.77 ms; ADC: 939.82 ± 83.81 × 10^−6^ mm^2^/sec vs. 992.40 ± 55.46 × 10^−6^mm^2^/sec; all *P* < 0.001) except for the PD value (79.90 ± 2.66 vs. 81.24 ± 3.24 pu; *p* = 0.065) (Table 3). T1 value showed equivalent diagnostic performance on STE prediction (AUC: 0.877, 95% CI: 0.777–0.943) to ADC (AUC: 0.737, 95% CI: 0.618–0.835; *p* = 0.081) while T2 value (AUC: 0.902, 95% CI: 0.807–0.960) possessed superior performance to ADC (*p* = 0.030). Furthermore, the combined T1 + T2 model had the best prediction efficacy (AUC: 0.982, 95%CI: 0.916–0.999) than ADC alone (*p* < 0.001) (Table 4, Fig. 6).

**Table 4:** Comparing the predictive performance of ADC, T1, T2 value only, and T1 + T2 models for RRF and STE in CC patients

		AUC (95% CI)	Sen	Spec	Acc	Cutoff	*P* value
RRF (Early-stage cervical cancer)	ADC	0.776 (0.636,0.881)	0.563	0.882	0.722	938.800	Ref
	T1	0.854 (0.725,0.938)	0.938	0.677	0.808	1258.667	0.338
	T2	0.908 (0.792,0.971)	0.813	0.882	0.848	93.333	0.121
	T1 + T2	0.980 (0.893,0.999)	0.100	0.941	0.971	0.263	0.005*
STE (Locally advanced cervical cancer)	ADC	0.737 (0.618,0.835)	0.500	0.941	0.721	926.967	Ref
	T1	0.877 (0.777,0.943)	0.778	0.941	0.860	1193.667	0.081
	T2	0.902 (0.807,0.960)	0.861	0.882	0.872	88.667	0.030*
	T1 + T2	0.982 (0.916,0.999)	0.917	0.971	0.944	0.430	< 0.001*

ADC, apparent diffusion coefficient; AUC, area under the curve; CI, confidence interval; Acc, accuracy; Spec, specificity; Sen, sensitivity; Ref, reference; STE, short-term efficacy; CCRT, concurrent chemoradiotherapy; RRF: recurrence risk factors

*p value indicates a statistical difference. *P* values were calculated using DeLong test

## Discussion

MAGiC is a novel imaging method that can offer both multiple contrast weighted imaging (sy-T2WI) as well as quantitative mapping (T1, T2 and PD maps) acquired with a two-dimensional fast spin echo multi-delay multi echo (MDME) technique in one scan within a clinically-acceptable scan time. In our study, sy-T2WI showed sufficient image quality and equivalent diagnostic performance on differentiation of tumor stage using conventional hr-T2WI as standard reference. Moreover, the combination model (T1 + T2 maps) was superior to ADC in predicting RRF and STE in patients with cervical cancer.

The primary challenge for synthetic morphologic imaging is image quality for clinical applications. Our findings demonstrated that sy-T2WI have comparable image quality scores of anatomical details, distortion, artifacts, and lesion conspicuity to hr-T2WI [31, 32]. Furthermore, the excellent intra-reader agreement of image quality scores was in line with the findings reported by Meng et al. [33] and inter-modality correlation in differentiating stage IB-IIA from IIB-IVA (k = 0.935; *p* < 0.001), implying both shared a similar diagnostic accuracy on T-stage rectal cancer [31]. sy-T2WI showed excellent accuracy (0.908), sensitivity (0.908) and specificity (0.999) in identifying lesions for surgery and CCRT using hr-T2WI as the standard reference. Overall, MAGiC is a reliable method for cervical lesion delineation, stage evaluation and strategy determination in delineating cervical lesions.

The mean T1, T2, PD and ADC values of myometrium were relatively lower to the low-end reported values [34, 35] due to the various MRI system. They were not significantly different between RRF and non-RRF group as well as between non-residual group and tumor residual group. However, quantitative T1 and T2 values in our study were significantly lower respectively in the RRF and non-residual group than in the non-RRF and tumor residual group, in consistent to previous studies [27, 31, 33, 36]. T2 transverse relaxation time is determined by the amount of free water, bound water, and macromolecules within the lesion [37]. The shortened T2 of cervical lesions may attribute to reduce water content, macromolecules and extracellular space (increased tumor proliferation), especially in poorly- differentiated tumors and those with RRF [38]. The poorly-differentiated tumors are more sensitive to CCRT, leading to complete remission [39]. T1 relaxation time depends on the precession frequency of the energy transfer from an excited proton to the surrounding molecules [40]. The reduction of extracellular space in the tumor tissue lowers down the precession frequency of molecules, shortening T1 value of the lesions in the non-residual and RRF groups. In spite that the PD value provides proton density for tissue water content, there was no difference between the residual and non-residual as well as RRF and non-RRF groups in our study and previous studies [22, 27, 31, 33]. The RRF evaluation in early-stage CC patient—such as LNM, LVSI, DSI and TMD status—is crucial to determine the aggressiveness of the malignant neoplasms, treatment management and prognosis. In accordance with the MAGiC-derived quantitative T1 and T2 for evaluation of the LVSI status in CC patients [41], those efficacy of post-treatment RRF still needs further validation. As a whole, the statistically T1 and T2 values could be reliable imaging biomarkers and had potential in predicting RRF and STE in CC patients.

DWI has been widely used for diagnosis and prognosis in CC patients [4, 9, 10, 42]. Consistent with previous findings [43–45], lower ADC values were observed respectively in the RRF and non-tumor residual groups compared to the non-RRF and tumor residual groups. This demonstrated that the pre-treatment ADC is valuable for evaluating RRF and therapeutic response [46, 47]. The ADC shared a similar diagnostic performance to T1 value for RRF and STE and T2 value for RRF but inferior to T2 value for STE. In other words, T2 value is more sensitive in assessing STE than RRF in consistent with previous reports [16, 27, 48–51]. Similar to previous findings [27, 48], the combined T1 + T2 model demonstrated superior diagnostic performance compared to ADC value, likely attributable to the excellent reliability and repeatability of MAGiC-derived T1 and T2 measurements [52, 53]. It implied that MAGiC provided enhanced capability for evaluating RRF and STE in CC patients relative to to DWI. Furthermore, the integration of multiple quantitative parameters (T1, T2 and PD value derived from MAGiC) may facilitate more precise treatment decision-making while potentially improving cost-effectiveness and accessibility for CC patients. These findings position MAGiC as a promising and reliable MR imaging technique for comprehensive tumor assessment.

This study had several limitations. First, the comparison of synthetic and traditional T1 and T2 mapping is not carried out due to an impractical design in clinical work (the scan time could be up to 30 min in one patient) and known several applications of MAGiC on brain, breast, rectus, prostate [21–27]. Second, three consecutive slices including the maximum cross-sectional lesion instead of the whole lesion for T1, T2 and PD measurements were performed, likely contributing to lost details from the resting part of lesions. Third, the CC patients were only confirmed by biopsy and has no pre-surgical information such as pathology data. However, the FIGO staging were evaluated only using hr-T2WI for investigating the feasibility of sy-T2WI in determination of a treatment plan. In future work, FIGO staging could be performed using both sy-T2WI and DWI for improving diagnosis accuracy. Forth, the sample size was relatively small in the subgroups to assess diagnostic efficacy of MAGiC on RRF and STE and the prospective study was performed in a single center. A larger cohort is warranted to generalize the results, including and the evaluation of LNM, LVSI, DSI, TMD status using quantitative maps, and multicenter data are conducted to avoid data bias.

In conclusion, MAGiC can generate synthetic morphologic (sy-T2WI) and quantitative images (T1, T2, and PD maps) in one acquisition. sy-T2WI possessed comparable image quality and equivalent accuracy of tumor staging to hr-T2WI. The average T1 and T2 values were significantly lower in the RRF and non-residual groups compared to the non-RRF and tumor residual groups. The T1 values has similar performance to ADC in predicting RRF and STE, while T2 value is superior to ADC for forecasting STE and similar to ADC for RRF. Furthermore, T1 combined with T2 is superior to ADC in predicting STE and RRF. Thus, MAGiC is a promising technique for guiding therapeutic planning and predicting RRF and STE in CC patients, and is similar and even superior to hr-T2WI and DWI. 

## Supplementary Information

Below is the link to the electronic supplementary material.

Supplementary Fig 1. Representative pathological images of lymph node metastasis (LNM), positive lymphovascular space invasion (LVSI), deep cervical stromal invasion (DSI) and tumor maximum diameter (TMD). **A** LNM: the lymph node was invaded by malignant epithelial cells (white arrows; HE staining, ×100) showed, **B** LVSI: arcinoma cells were shown in tumor microvessels (white arrows; HE staining, ×100), **C** DSI: tumor invasion greater than one-third of the cervical wall (white arrows; HE staining, ×100), and **D** TMD: the longest diameter of mass was more than 4cm (white arrows; HE staining, ×100) (TIF 36631 KB)

Supplementary file2 (DOCX 13 KB)
