# Supplementary figures and images for: Assessment of magnetic resonance image compilation (MAGiC) abilities of therapeutic selection and prediction on recurrence risk factors and short-term treatment efficacy in cervical cancer
Source: Radiol Med. 2025 Jul 4;130(9):1325–38. doi: 10.1007/s11547-025-02042-7 (PMC12454491; doi:10.1007/s11547-025-02042-7)

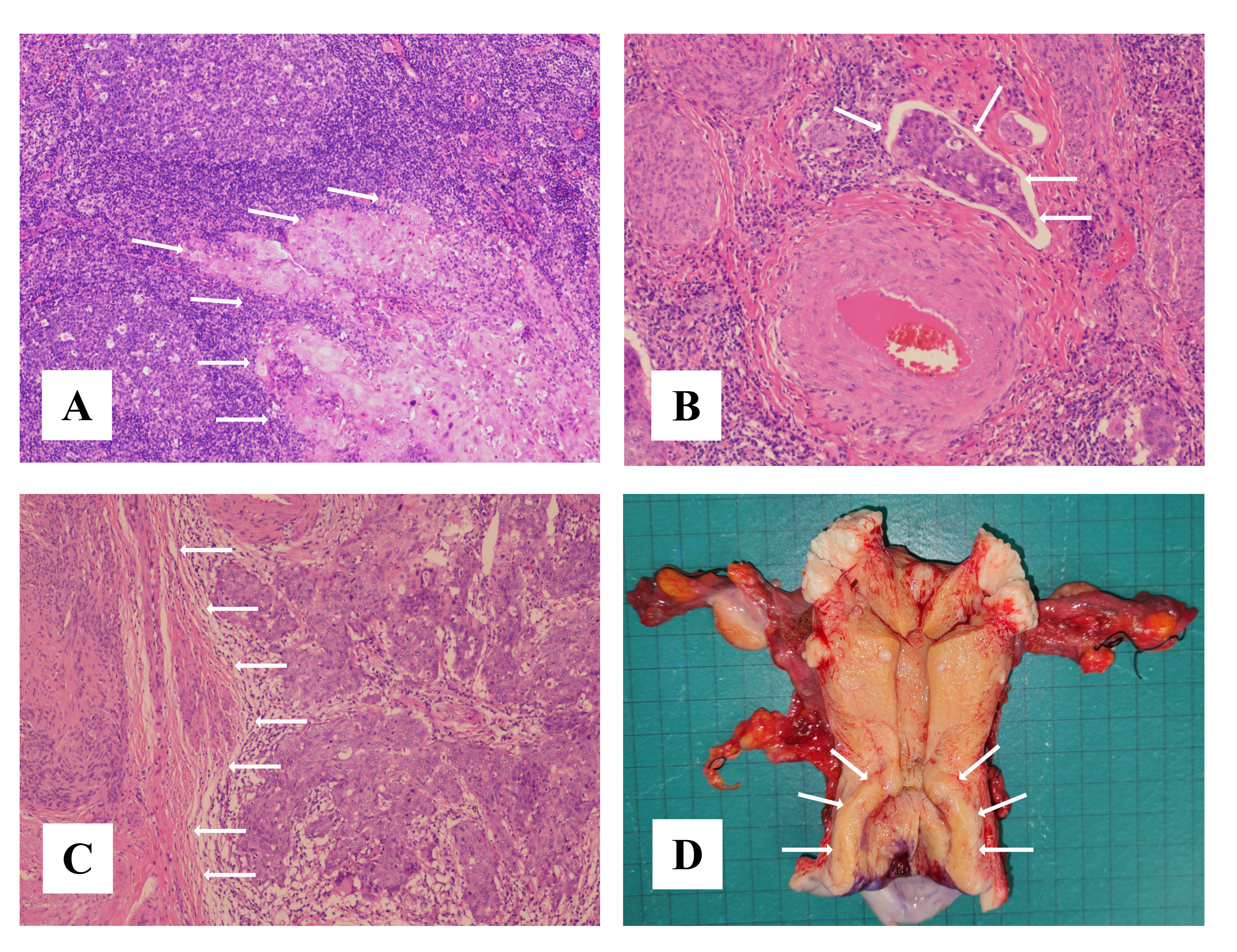

Supplement: Supplementary file 1 — Supplementary Fig 1. Representative pathological images of lymph node metastasis (LNM), positive lymphovascular space invasion (LVSI), deep cervical stromal invasion (DSI) and tumor maximum diameter (TMD). A LNM: the lymph node was invaded by malignant epithelial cells (white arrows; HE staining, ×100) showed, B LVSI: arcinoma cells were shown in tumor microvessels (white arrows; HE staining, ×100), C DSI: tumor invasion greater than one-third of the cervical wall (white arrows; HE staining, ×100), and D TMD: the longest diameter of mass was more than 4cm (white arrows; HE staining, ×100) (TIF 36631 KB) [file 11547_2025_2042_MOESM1_ESM.tif]
