# Supplementary material for: Assessment of magnetic resonance image compilation (MAGiC) abilities of therapeutic selection and prediction on recurrence risk factors and short-term treatment efficacy in cervical cancer
Source: Radiol Med. 2025 Jul 4;130(9):1325–38. doi: 10.1007/s11547-025-02042-7 (PMC12454491; doi:10.1007/s11547-025-02042-7)
Supplement: Supplementary file 2 — Supplementary file2 (DOCX 13 KB) [file 11547_2025_2042_MOESM2_ESM.docx]

| Supplementary table 1, The patient clinical characteristics. | |
| --- | --- |
| Characteristics Value |  |
| Total Patients | 194 |
| Median age (years) | 52.4 ± 9.2 |
| Age range (years) | 26 - 84 |
| Included patients | 119 |
| Median age (years) | 50.4 ± 10.0 |
| Age range (years) | 26-80 |
| Max tumor diameter (cm) | 2.3-54.7 |
| FIGO stage by hr-T2WI |  |
| IB-IIA for surgery (early-stage CC patients) | 50 |
| RRF group | 16 |
| No-RRF group | 34 |
| IIB-IVA for CCRT (advanced CC patients) | 69 |
| Tumor residual group | 36 |
| non-residual group | 33 |
| Histologic types |  |
| Squamous cell carcinoma | 106 |
| early-stage CC patients | 41 |
| advanced CC patients | 63 |
| Adenocarcinoma | 15 |
| early-stage CC patients | 9 |
| advanced CC patients | 6 |
| Grades |  |
| Well/moderate differentiation | 104 |
| early-stage CC patients | 46 |
| advanced CC patients | 58 |
| Poorly differentiation | 15 |
| early-stage CC patients | 4 |
| advanced CC patients | 11 |
